# Single-cell transcription analysis reveals the tumor origin and heterogeneity of human bilateral renal clear cell carcinoma

**DOI:** 10.1515/biol-2022-0569

**Published:** 2023-02-09

**Authors:** Zhengqiang Wan, Yinglei Wang, Aiqun Li, Cheng Li, Dongbing Zheng

**Affiliations:** The Second Clinical Medical College of Binzhou Medical University, Shandong, China; The Second Ward of Urology, Yantai Affiliated Hospital of Binzhou Medical University, Shandong, China; Emergency Surgery, Yantai Affiliated Hospital of Binzhou Medical University, Shandong, China

**Keywords:** single-cell transcriptome, bilateral renal clear cell carcinoma, intratumoral heterogeneity, tumor microenvironment, cellular differentiation trajectory

## Abstract

Bilateral renal clear cell carcinoma (BRCC) is a rare type of renal cell carcinoma (RCC) that accounts for only 1–5% of RCC cases and has a poor clinical prognosis. The origin, tumor microenvironment, cellular molecular features, and intra-tumoral heterogeneity of BRCC are still unclear. We downloaded BRCC single-cell transcriptome sequencing data from the gene expression omnibus database biochip GSE171306, containing 3,575 cells from left-sided clear cell renal cell carcinoma (ccRCC) and 3,568 cells from right-sided ccRCC, and used a series of R packages for data quality control (QC) and subsequent analysis of BRCC single-cell transcriptome data, including the use of the R packages Seurat and scCancer for cell QC, identification of major cell types, and cell annotation; R package scran for calculation of cell cycle scores; R package infercnv for malignancy scoring of tumor cells; R package ReactomeGSA for functional enrichment analysis; R package Monocle 2 for the analysis of cell differentiation trajectories; and R package CellphoneDB for the analysis of intercellular interactions. In this study, by analyzing the high-quality single-cell transcriptome data of BRCC, we identified 18 cell types and found that left- and right-sided ccRCC were approximately the same in terms of cell type and the number of each cell but differed significantly in terms of tumor cell malignancy score, tumor microenvironment, and cell stemness score. In the cell differentiation trajectory analysis of BRCC, we found that endothelial cells and macrophages play an extremely important role in its tumor progression. Further cell communication analysis was performed, and we found that it may signal through ligand–receptors, such as vascular endothelial growth factor–vascular endothelial growth factor receptor1 (VEGF–VEGFR1), MIF–(CD74-CXCR4), and growth arrest-specific protein 6–AXL, to influence the development of BRCC. The analysis of single-cell transcriptomic data of human BRCC suggests that left- and right-sided ccRCC may be of the same tumor origin, but the left-sided ccRCC is more malignant and has a better immune response.

## 1 Introduction

Renal cell carcinoma (RCC) is a common malignancy of the urinary tract, usually originating from renal tubular epithelial cells. RCC is divided into several histopathological subtypes, including clear cell renal cell carcinoma (ccRCC), papillary RCC (pRCC), suspicious RCC (chRCC), and other rare subtypes, accounting for 80, 10–15, and 4–5% of RCC, respectively [1,2]. BRCC is clinically rare, accounting for only 1–5% of all kidney cancer patients [3,4,5]. BRCC is classified according to the time of diagnosis of bilateral tumors as concurrent BRCC, where the time interval between diagnosis of bilateral renal tumors is ≤6 months, and heterochronic BRCC, where the time interval between diagnosis is >6 months [6,7]. Moreover, 84–95% of BRCC patients have the same bilateral pathology, with renal clear cell carcinoma being the predominant pathological type [8,9]. Clinically, metastatic and non-metastatic renal carcinoma are the most common types. Clinically, metastatic and non-metastatic renal cancers have very different treatment strategies and follow-up regimens, making the study of the mechanisms of tumor development in BRCC of clinical importance [10]. The study of the mechanisms of BRCC tumor development is therefore of great clinical importance. To date, the origin of BRCC tumors has not been elucidated, and there is still a considerable debate as to whether both tumors are primary or whether one tumor is primary and the other metastatic [7]. Kito et al. [11] analyzed the tumor genetic characteristics of eight patients with BRCC and showed that five cases of heterozygous BRCC had the same chromosomal allele deletion, suggesting that the bilateral tumors of heterozygous BRCC had the same clonal origin; two cases of simultaneous BRCC had the same allele deletion locus and one case was different, suggesting that there were two types of tumor clonal evolution in simultaneous BRCC, dual primary and contralateral metastasis. In a multicenter prospective study, whole-exome sequencing of tumor tissues was performed in 101 ccRCC patients, with three tumors occurring bilaterally, and the results showed that the tumors in these three patients were not of the same origin on both sides [12]. Kume et al. [13] and Ji et al. [14] also showed heterogeneity in the origin of tumors in BRCC. The clinical incidence of BRCC is low, but the prognosis of patients is poor. Studying this disease at the single-cell level will help to further understand the cellular molecular features, tumor origin, and heterogeneity of this tumor and provide new references for its clinical treatment and molecular mechanisms.

## 2 Materials and methods

### 2.1 Data sources

We obtained 3,575 and 3,568 single-cell transcriptomic data for left- and right-sided renal clear carcinoma tissue samples from the biochip GSE171306 through the Gene Expression Omnibus database (https://www.ncbi.nlm.nih.gov/geo/).

### 2.2 Single cell transcriptome sequence (scRNA-Seq) data processing and cell type identification

The data for this study were obtained from public data provided by GSE171306 cells with min.nUMI ≤ 500 and max.nUMI ≥ 20,000 and total number of genes min.nGene ≤ 200 and max.nGene ≥ 6,000 were filtered out using R package Seurat version 3.2.2 for cell quality control (QC), and further filtered out cells with percentage of mitochondrial genes >37.5%, ribosome gene percentage >44.1% and dissociation-associated gene percentage >9.80% (Figure S1a), and a total of 6,063 left-sided ccRCC cells and 4,944 right-sided ccRCC cells were obtained (Figure S1b). The post-QC data were normalized using the R package Seurat (version 3.2.2), and data were integrated using principal component analysis, t-distributed stochastic neighbor embedding (t-SNE), and uniform manifold approximation and projection (UMAP) for left- and right-sided ccRCC scRNA-Seq data for subsequent analysis (Figure S1c). To ensure the reliability of the data, we further calculated the doublet score for both samples (Figure S1d and e) to ensure the usability of the data. After performing rigorous QC, we used cell type markers for cell annotation and t-SNE and UMAP for reduced dimensional clustering.

### 2.3 Cell malignancy, cell cycle, and cell stemness scores

To identify malignant and non-malignant cells in BRCC cells and assess the cell cycle and cell stemness of BRCC cells, we obtained BRCC scRNA-Seq data, rated malignant and non-malignant cells based on copy number alterations (CNV) using the R package infercnv, assigned cell cycle-related scores to each cell based on G2/M and S-phase gene expression, and calculated cell cycle using the CellCycle.score function in the R package Seurat version 3.2.2. Cell stemness was scored using the Stemness. score function in the R package Seurat, version 3.2.2.

### 2.4 Cell type annotation and tumor microenvironment

To investigate whether there are differences in the tumor microenvironment between left- and right-sided ccRCC, we annotated the BRCC tumor microenvironment cell types separately using a one-class logistic regression model, with cell types including endothelial cells, fibroblasts, and immune cells (CD4+ T cells, CD8+ T cells, B cells, natural killer cells, and bone marrow cells).

### 2.5 Functional enrichment analysis

Reactome (Home-Reactome Pathway Database) is the open-source enrichment analysis database [15]. It documents the relationship between signaling and metabolic molecules, as well as their constituent biological pathways and processes. Pathway significant enrichment analysis uses the Reactome Pathway as a unit and applies hypergeometric tests to identify pathways that are significantly enriched in differentially expressed genes compared to the whole genomic context. ReactomeGSA (https://github.com/reactome/ReactomeGSA) is a commonly used R package for functional enrichment analysis of scRNA-Seq data. Here, we use the R package ReactomeGSA to analyze the functional enrichment of each cellular subtype and explore the biological pathways through which each BRCC scRNA-Seq cellular subtype functions.

### 2.6 Exploring the characteristics of BRCC cell differentiation trajectories

Monocle (https://bioconductor.org/packages/release/bioc/html/monocle.html) is currently the most commonly used R package for assessing cell differentiation trajectories [16]. It uses independent component analysis to project all cells in a low-dimensional space (usually two-dimensional); next, a minimal spanning tree is used to connect the major cell points to form a “skeleton” of the differentiation trajectory, and then all remaining cells are projected onto the “skeleton”. This dimension is called pseudo-time and represents the predicted genealogical trajectory of the cells in the sample [17]. Here, we use the R package Monocle2 to infer the cell differentiation trajectory of BRCC to explore the differences, origins, and heterogeneity of BRCC cell development.

### 2.7 Interaction patterns between BRCC cells

Ligand–receptor complex-mediated cellular communication is essential for coordinating a wide range of biological processes, such as development, differentiation, and infection responses. CellphoneDB (https://pypi.org/project/CellPhoneDB/) is a repository of ligands, receptors, and their interactions [18], whose database takes into account the subunit structure of ligands and receptors and accurately represents heterodimeric complexes, enabling a comprehensive and systematic analysis of intercellular communication molecules. To analyze ligand–receptor interaction relationships between various cell types in the BRCC tumor microenvironment, we used the R package CellChat to calculate ligand–receptor interaction scores between cell subtypes. CellChat uses a large number of action laws by combining gene expression with previously known knowledge of the interactions between signaling ligands, receptors, and their cognate factors to make use of the cell–cell communication is modelled by assigning each interaction with a probability value and performing alignment tests to infer biologically meaningful cell–cell communication.


**Ethical approval:** This study was approved by the Ethics Committee of Binzhou Medical University. The samples used in this study were taken from public databases and no informed consent was required from the patients.

## 3 Results

### 3.1 Left- and right-sided ccRCC of the same tumor origin and analysis of inter- and intratumoral heterogeneity and functional enrichment in BRCC

**Figure 1:**
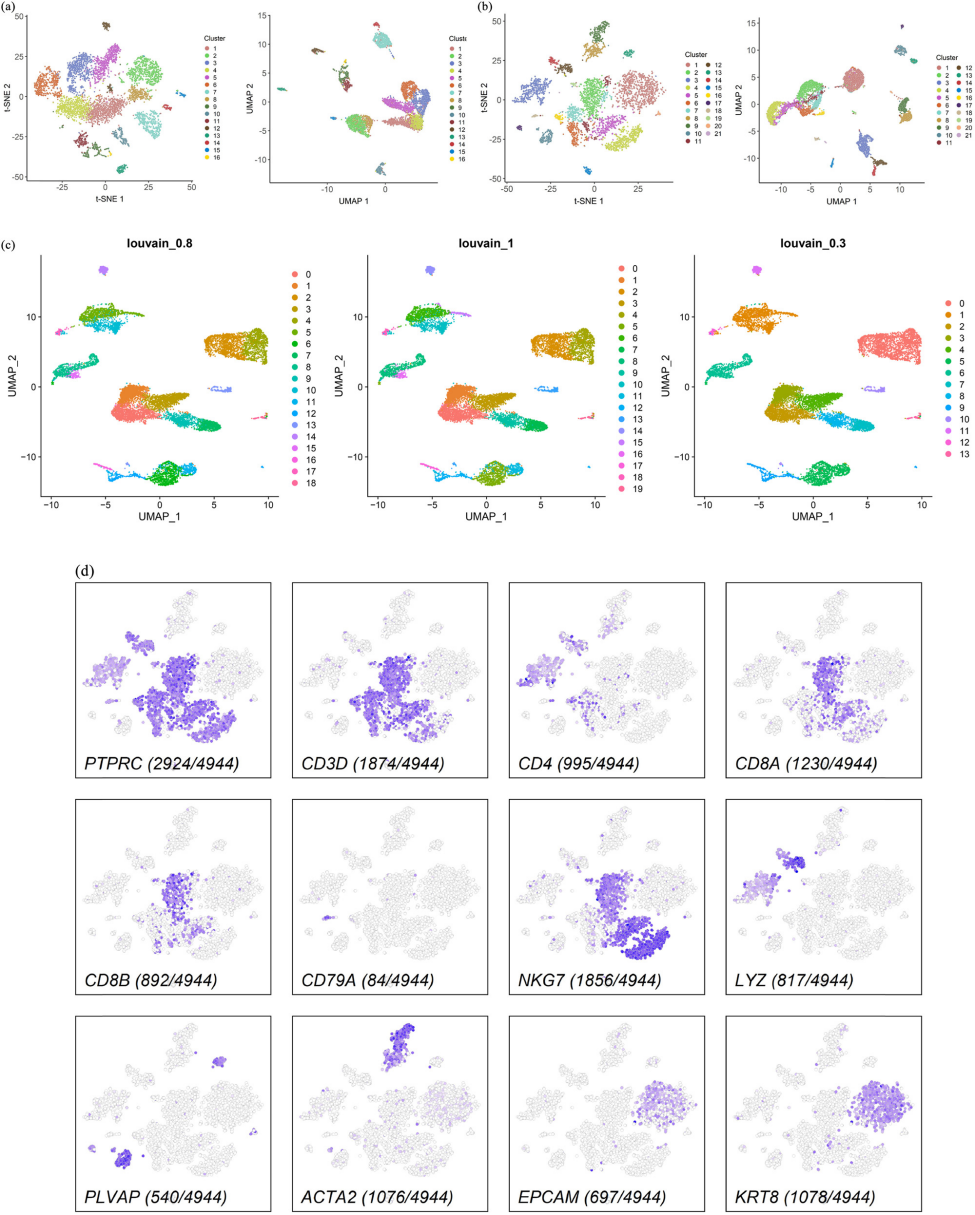

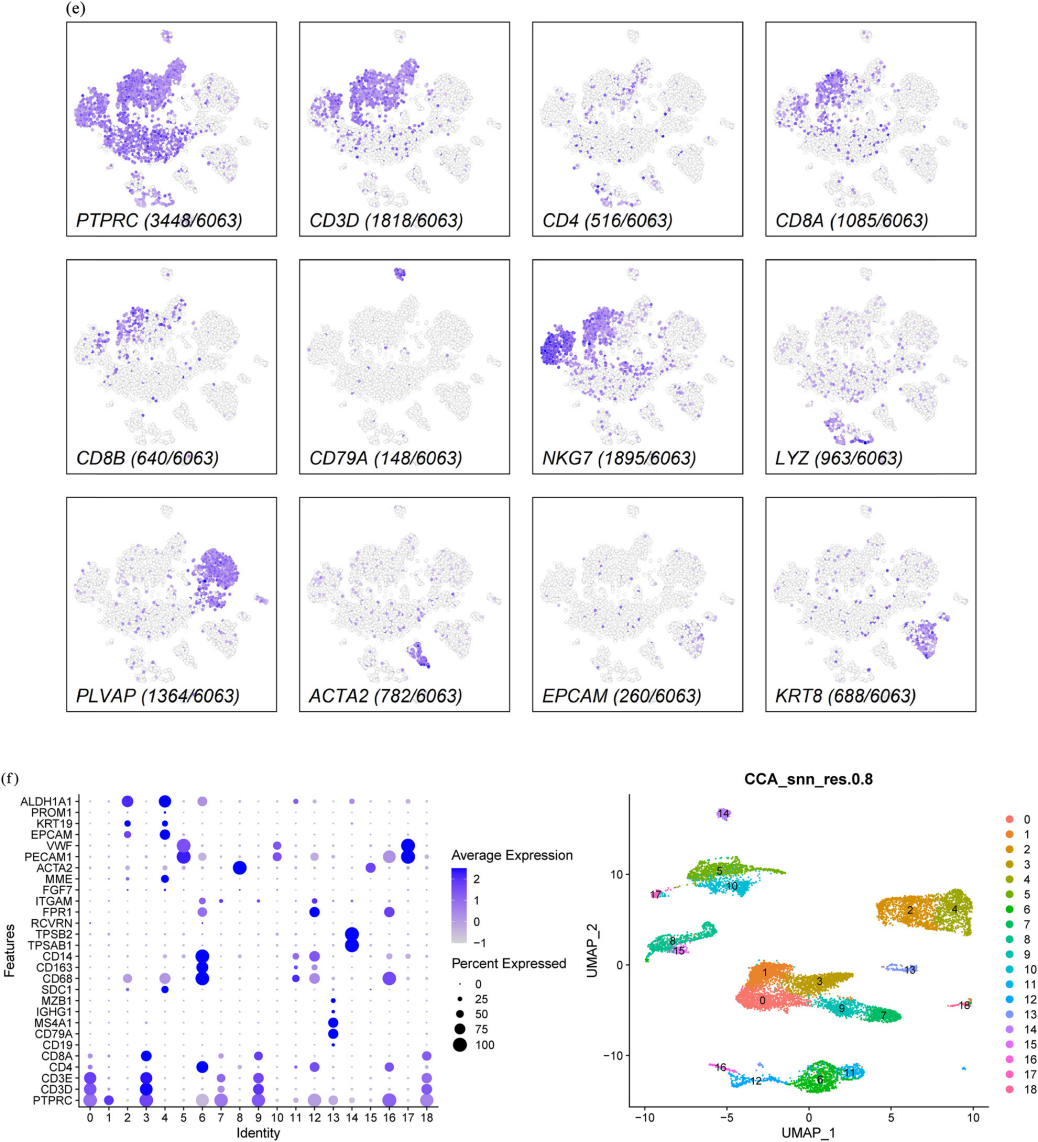

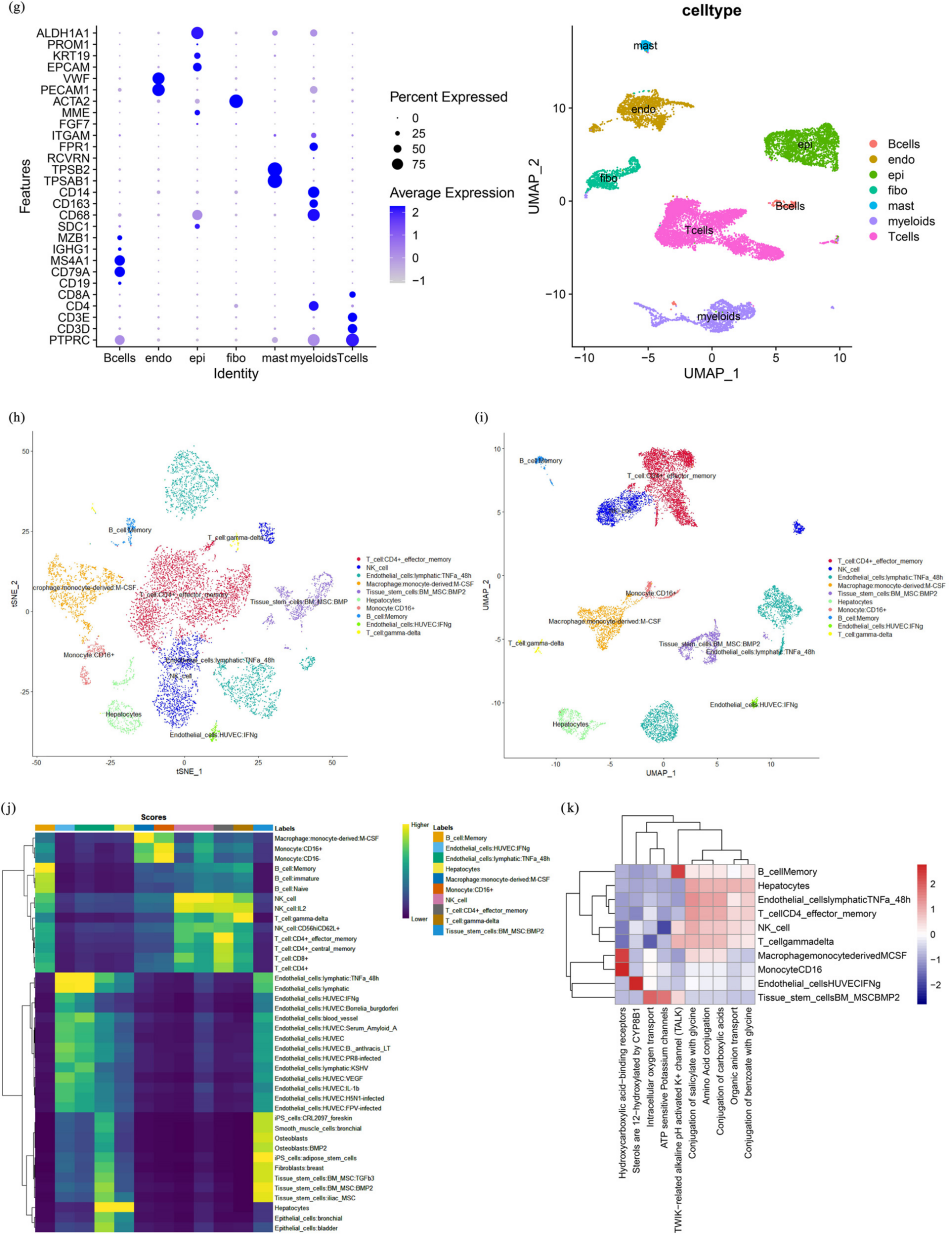
Single-cell transcriptomic map of sporadic BRCC. (a) t-SNE and UMAP plot representation of left-sided ccRCC with 16 distinct cell clusters. (b) t-SNE and UMAP plot representation of right-sided ccRCC with 21 distinct cell clusters. (c) UMPA clustering of BRCC by different dim values. (d) Expression of marker genes for the cell types in right-sided ccRCC. (e) Expression of marker genes for the cell types in left-sided ccRCC. (f) Expression of marker genes for the cell types in BRCC. (g) UMPA clustering of BRCC by cell type. (h) Cell annotation for BRCC with UMAP dimensionality reduction. (i) Cell annotation for BRCC with t-SNE dimensionality reduction. (j) A heatmap of the correlation between each cell type in the BRCC and the reference cell type in the Human Primary Cell Atlas (HPCA). (k) Pathway enrichment analysis of BRCC.

It is unclear whether the sporadic BRCC tumor cells are of the same origin, so we proposed to investigate the origin of sporadic BRCC tumor cells at the single-cell level and further clarify whether there is intra- and intertumoral heterogeneity in BRCC. To this end, we first performed dimensional clustering using t-SNE and UMAP on the left- and right-sided ccRCC scRNA-Seq data after QC, respectively, with dim set to 0.8. The left-sided ccRCCs were clustered into16 cell clusters and the right-sided ccRCCs were clustered into 21 cell clusters (Figure 1a and b). Based on the characteristics of BRCC integrated data after QC, we chose UMAP for non-linear dimensionality reduction and clustered BRCC into 18, 19, and 13 cell clusters by setting dim values to 0.8, 1.0, and 0.3, respectively. In view of the above analysis results, we believe that setting dim to 0.8 can better identify the cell types of BRCC and subsequent BRCC. All subsequent analyses of the BRCC data were carried out with a dim value of 0.8 and 18 cell clusters (Figure 1c). To clarify the cell types of the two samples, we used cell type markers for cell annotation of the cells after QC of the two samples, and the cell type markers are referred to in Table 1. By cell annotation, we found that the highest proportion of cells in both the left- and right-sided ccRCC were T cells (3448/6068 on the left-sided and 2924/4944 on the right-sided), followed by natural killer (NK) cells (1895/6068 on the left-sided and 1856/4944 on the right-sided), and the proportion of cells in both related cell types was approximately the same (Figure 1d and e), which, together with the previous clustering of cells in both, suggests that the origin of the left- and right-sided ccRCC tumor cells may be of the same origin. In order to explore the tumor microenvironment of BRCC, such as T cells, B cells, endothelial cells, fibroblasts, epithelial cells, stromal cells, and mast cells, we used molecular markers of the above cells for cell annotation. We found that T cells (including CD4+ T cells and CD8+ T cells) were the cell type with the highest number of BRCC cells, followed by epithelial cells. The above revealed the complex internal environment of BRCC (Figure 1f and g). To calculate the characteristics of each of the 18 cell clusters in BRCC, we performed differential expression analysis by running the Seurat V3.2 function to extract the expression of the TOP3 gene that was most differentially expressed in each cell cluster and plotted it as a bubble plot, and we found a clear linear relationship between cell cluster and TOP3 gene expression (Figure S2). To further identify the cell types of BRCC, we cell annotated BRCC with reference to the cell type markers of HPCA and performed UMAP and t-SNE clustering to explore the cell types it contained. We found that BRCC contained a total of ten cell types, and peculiarly, we found a small number of hepatocytes in BRCC. To study the pathological mechanism of BRCC, we used the R package ReactomeGSA to explore the signaling pathways associated with its progression. Here, we used a heat map to demonstrate the pre-T0P ten signaling enrichment pathway, and we found that its tumor progression was mainly associated with metabolism-related pathways, such as hydroxycarboxylic acid-binding receptors, benzoate, and glycine binding, and amino acid and carboxylic acid conjugation (Figure 1k).

**Table 1:** Cell type classical markers

Cell type	Markers
T cells	PTPRC, CD3D, CD3E, CD8A, and CD8B
T cells (CD4+)	PTPRC, CD3D, and CD4
T cells (CD8+)	PTPRC, CD3D, CD8A, and CD8B
B cells	PTPRC and CD79A
NK cells	PTPRC, NKG7, FGFBP2, FCG3RA, and CX3CR1
Myeloid cells	PTPRC and LYZ
Endothelial cells	PLVAP, PECAM1, and VWF
Fibroblast cells	ACTA2, FGF7, and MME
Epithelial cells	EPCAM, KRT8, KRT19, PROM1, and ALDH1A1
Monocytes and macrophages cells	CD68, CD163, and CD14
Plasma cells	IGHG1, MZB1, SDC1, and CD79A
Stromal cells	CD10+, MME, CD31+, and PECAM1
Photoreceptor cells	RCVRN

### 3.2 Differences in cell malignancy score, cell cycle score, cell stemness score, and tumor microenvironment were found between left- and right-sided ccRCC

**Figure 2:**
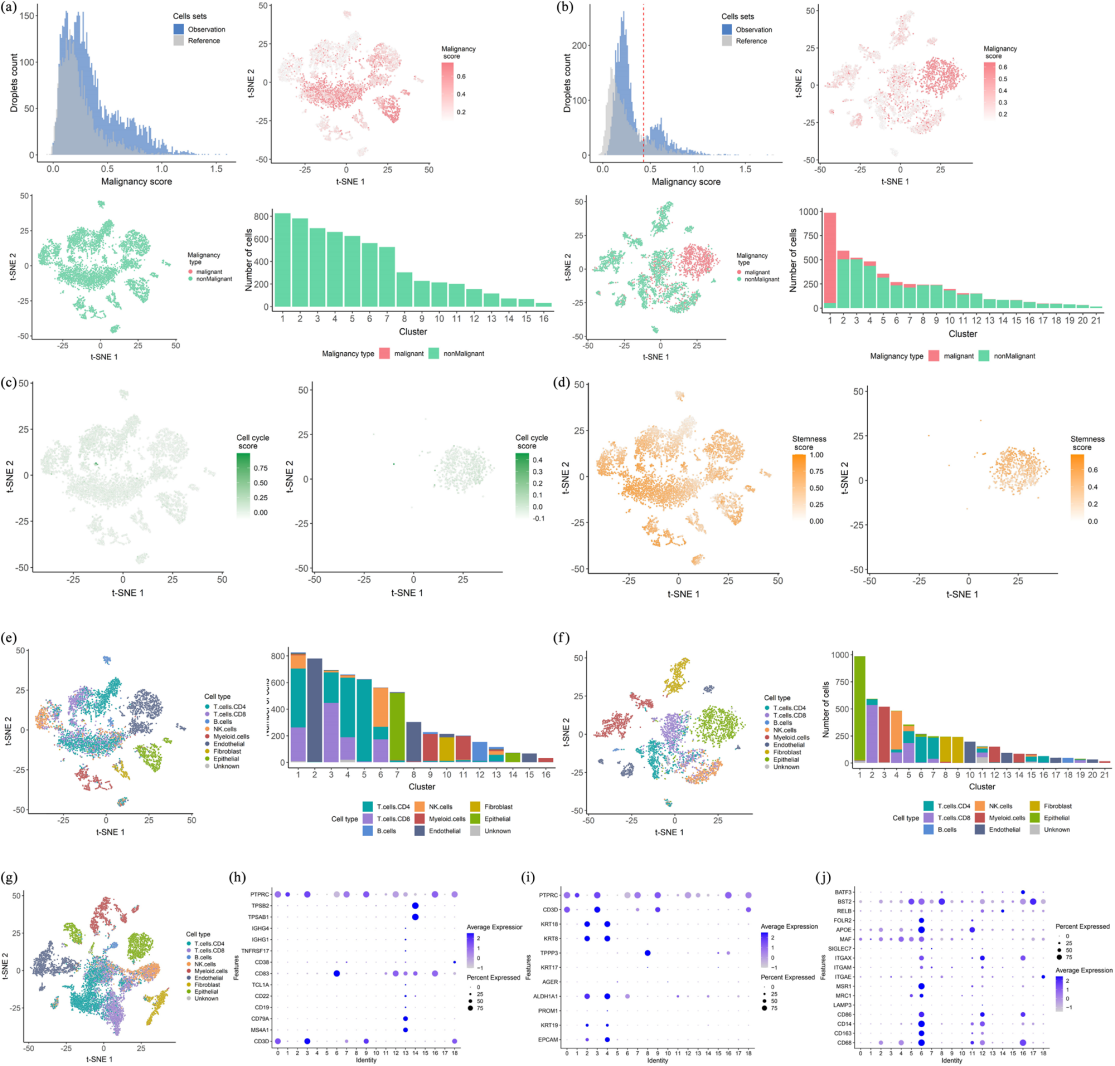
(a) Cell malignancy estimation of the left-sided ccRCC. (b) Cell malignancy estimation of the right-sided ccRCC. (c) Cell cycle estimation of left-sided ccRCC (left-sided) and right-sided ccRCC (right-sided). (d) Cell stemness estimation of left-sided ccRCC (left-sided) and right-sided ccRCC (right-sided). (e) Tumor microenvironment cell type annotation in left-sided ccRCC. (f) Tumor microenvironment cell type annotation in right-sided ccRCC. (g) t-SNE plot representation of tumor microenvironment cell type annotation in BRCC. (h) A bubble diagram showing B cell marker gene expression levels in different cell clusters of BRCC. (i) Epithelial cell marker gene expression levels in different cell clusters of BRCC. (j) A bubble diagram showing myeloid cell marker gene expression levels in different cell clusters of BRCC.

To assess the degree of malignancy of BRCC cells, we scored the cell malignancy of BRCC scRNA-seq data after QC based on CNV expression profiles and with the help of the R package infercnv. In the malignancy distribution map, we found that the distribution of the malignancy score of the left-sided ccRCC cells was not bimodal, and their cell malignancy score was higher after t-SNE downscaling. Based on the descending clustering of t-SNE, we found that the number of malignant cells in this sample was zero, and no malignant cells were found in its 16 cell subtypes. The percentage of malignant cells in each of the 16 cell clusters in its t-SNE downscaling was also extremely low (Figure 2a). In contrast to the malignancy score of the left-sided ccRCC cells, the malignancy score of the right-sided ccRCC cells was significantly different. We found that the distribution of the malignancy score of the right-sided ccRCC cells was clearly bimodal, with a bimodal boundary close to 4.29 × 10^−1^, and their malignancy score was higher after t-SNE downscaling than that of the left-sided, which was based on the type of malignancy (malignant and non-malignant). After the t-SNE downscaling, it can be found that some of the cells in its sample are malignant cells, and among the 22 cell clusters in its t-SNE downscaling clustering, malignant cells are present in several cell clusters, and its malignant cells are mainly concentrated in cell cluster 1 (Figure 2b). To investigate the cell cycle of BRCC tumor cells, we used the CellCycle.score function in the R package Seurat version 3.2 to calculate the possible cell cycle scores of each cell based on the expression of the classical marker genes in the G2/M and S phases. We found that the majority of cells in both the left- and right-sided ccRCC had a cell cycle score of 0, indicating G1 phase, and a small number of cells had a cell cycle score greater than 0, indicating S phase. In addition, some cells in the right-sided ccRCC had a cell cycle score less than 0, indicating G2M phase (Figure 2c). Stemness refers to the potential for normal cells to differentiate from their cells of origin into the various cell types that make up the human organism [19], and studies have shown that the progressive loss of cell differentiation and the acquisition of stem cell-like features are the main drivers of tumor progression [20,21]. To assess the stemness characteristics of BRCC cells, we used the Stemness.score function in the R package Seurat, version 3.2, to calculate their cell stemness scores. We found that the majority of cells with a stemness score greater than 0.5 in left-sided ccRCC and fewer cells with a stemness score greater than 0.5 in right-sided ccRCC suggest that left-sided ccRCC tumor cells are more prone to infiltrate and metastasize than right-sided ccRCC tumor cells (Figure 2d). Taken together, we suggest that there are differences in the biobehavioral of left- and right-sided ccRCC, with left-sided ccRCC being more malignant and more prone to progressive metastasis than right-sided ccRCC. To explore the complex tumor microenvironment of BRCC, with cell types including endothelial cells, fibroblasts, and immune cells (CD4+ T cells, CD8+ T cells, B cells, natural killer cells, and bone marrow cells), we calculated the expression levels of marker genes associated with each cell type in each cell cluster and thus inferred the distribution of each cell subtype of BRCC in its various cell clusters. We found that the cell types in the tumor microenvironment of left- and right-sided ccRCC were the same, both containing six cell types: endothelial cells, fibroblasts, T cells, B cells, natural killer cells, and myeloid cells, but the composition of the cell types in their tumor microenvironment was not the same. The most predominant cell type in the tumor microenvironment of the left-sided ccRCC was T cells (including CD4+ T cells and CD8+ T cells), whereas the most predominant cell type in the tumor microenvironment of the right-sided ccRCC was epithelial cells (Figure 2e and f), suggesting to some extent that there may be a difference in the response to immunotherapy between the left- and right-sided ccRCC and that the left-sided ccRCC may have a better immune response than the right-sided ccRCC. In the cell type annotation of the tumor microenvironment of BRCC, we found that T cells were the most predominant cell type, and by further calculating the distribution of each cell type in its 18 cell clusters, we could also find that T cells were highly expressed in all cell clusters, especially in the third and seventh cell clusters (Figure 2g and h). The expression levels of different cell types in different cell clusters were significantly different, with B cells being expressed at the highest level in cell cluster 14 (Figure 2i) and epithelial cells at the highest level in cell cluster 4 (Figure 2j). The above analysis suggests that there is a complex tumor microenvironment in BRCC, with significant heterogeneity between the left- and right-sided ccRCC tumor microenvironments, which could explain the differences in immunotherapy, drug resistance, and biological behavior between the two, as well as significant intratumoral heterogeneity in BRCC, which may explain the intratumoral differences in immune response and drug resistance in BRCC.

### 3.3 Characterization of BRCC cell developmental trajectories and dynamic changes revealed by Pseudotime

**Figure 3:**
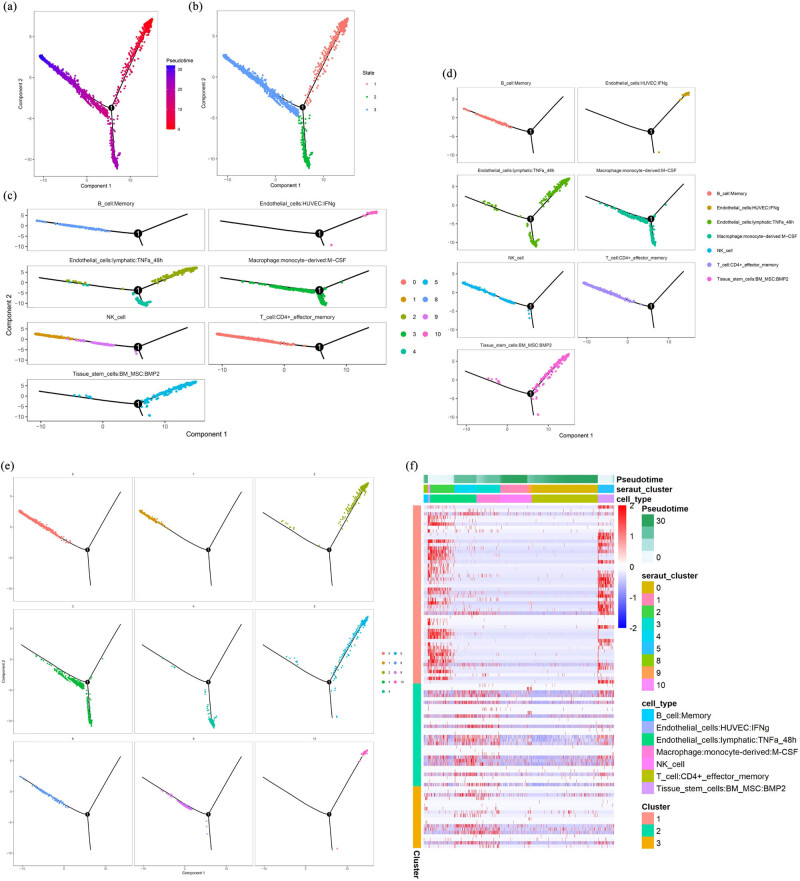
(a) t-SNE diagram of cell cluster distribution in the principal components of BRCC. The cells are sorted according to the pseudo-time value, from red to blue. (b) t-SNE diagram of cell cluster distribution in the principal component. Different colors represent different cell fate branches of the cell population. (c) According to the cell type, it is mapped to the sort of Pseudotime. (d) Differentiation status of each cell subtype. (e) According to the cluster ID of Seurat, it is mapped to the sort of Pseudotime. (f) The heatmap shows the branches in single-cell trajectories of BRCC.

To explore the developmental trajectory characteristics of BRCC, we used the R package Monocle 2 for analysis (Pseudotime). Here, we used Monocle 2’s “differentialGeneTest” function to filter the top 1,000 genes of clustering_DEG_genes as the feature gene set, its “Classifying cells by type” function for cell classification, its “DDRTree” function for dimensionality reduction, and its “BEAM” function for finding key branches. In the end, we obtained seven cell types, 11 cell clusters, and three key branches. When the trajectories were analyzed by cell type, we found that the seven cell types were in different states of differentiation. The majority of cells in B_cell:memory, T_cell:CD4+_effector_memory and Endothelial_cells:HUVEC are in the primitive state of cell differentiation. Endothelial_cells:lymphatic and Macrophage:monocyte-derived 2 cell types are actively differentiated and contain three states: primary, intermediate, and end-state. In addition, Endothelial_cells contained two subtypes of lymphocyte origin and vascular cell origin, with completely different differentiation trajectories, revealing the heterogeneous origin of BRCC. In analyzing the differentiation of the seven cell types in the ten cell clusters, we found that Endothelial_cells:lymphatic and NK_cell were actively differentiated, present in multiple cell clusters and in different differentiation states, and that there were differences in the differentiation state of the cells within the same cell cluster. For example, in Macrophage:monocyte, some cells are in the intermediate state and some are in the final state (Figure 3a–d). When trajectories were analyzed by cell clustering, we found that different cell clusters were in different states of cell differentiation, with the majority of cells in clusters 0, 1, 4, 8, and 9 in the terminal state, the majority of cells in cluster 10 in the primary state, and cells in clusters 2, 3, and 5 in the actively differentiated state, including the primary, intermediate, and terminal states (Figure 3e). In order to better understand the changes in BRCC, we explored the cell differentiation trajectories in “Pseudotime,” “cell_type,” and “seraut_cluster.” We found that most of the cells were in the end-state, and the majority of them were in the end-state. We found that most of the cells in BRCC were in the terminal state and had three main branches of differentiation, with branch I being the most actively differentiated, cell cluster 2 and cell cluster 5 cells being the most actively differentiated, and Endothelial_cells:lymphatic-derived and Macrophage:monocyte-derived being the two most active cell types. The cell types that play a key role in BRCC tumor progression are Endothelial_cells:lymphatic-derived and Macrophage:monocyte-derived, and the cell clusters that play a key role are cell cluster 2 and cell cluster 5 (Figure 3f). The above analysis partly reveals the characteristics of the cell developmental trajectory of BRCC.

### 3.4 Ligand–receptor analysis of BRCC reveals its intercellular interaction pattern

**Figure 4:**
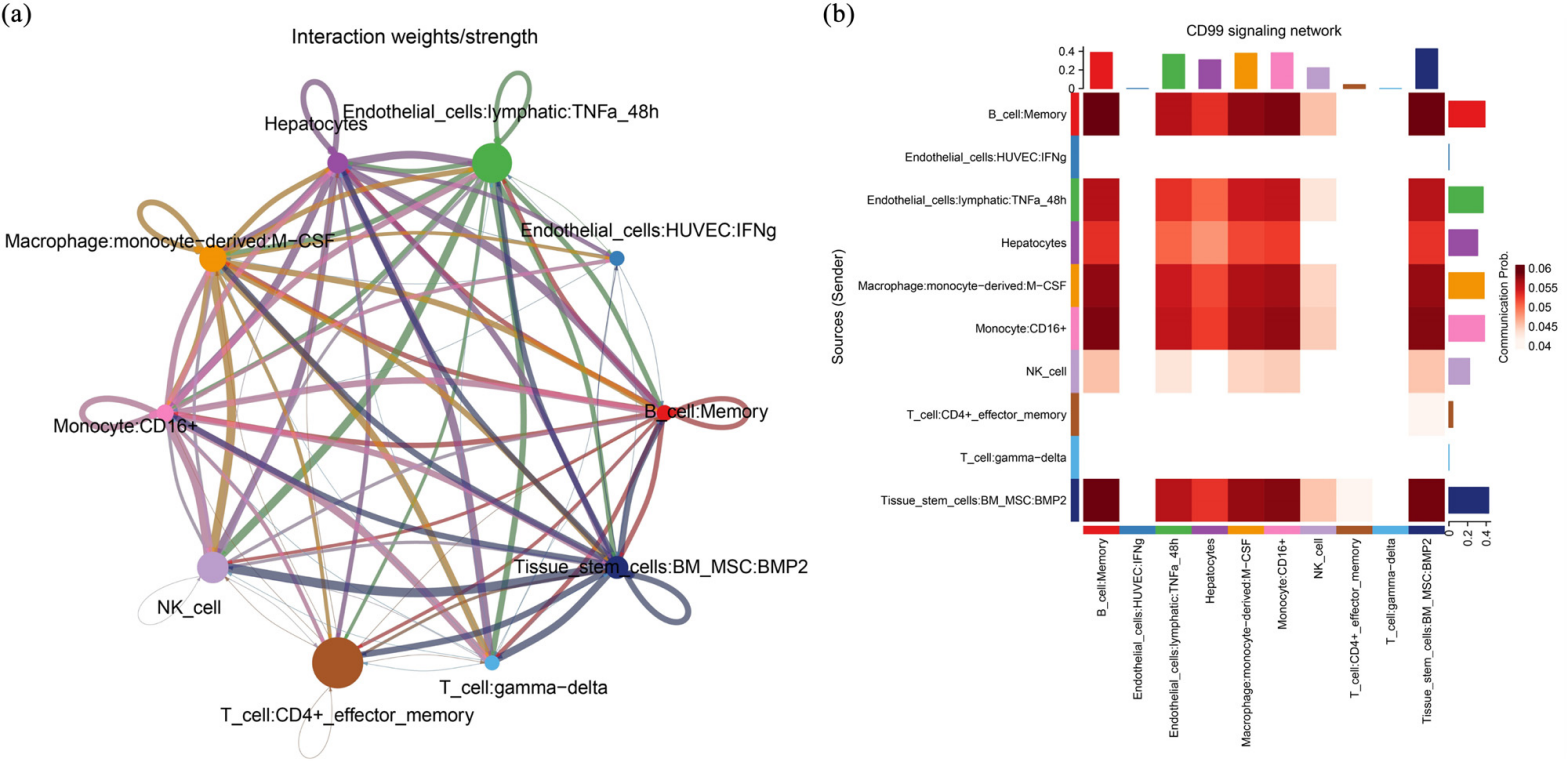

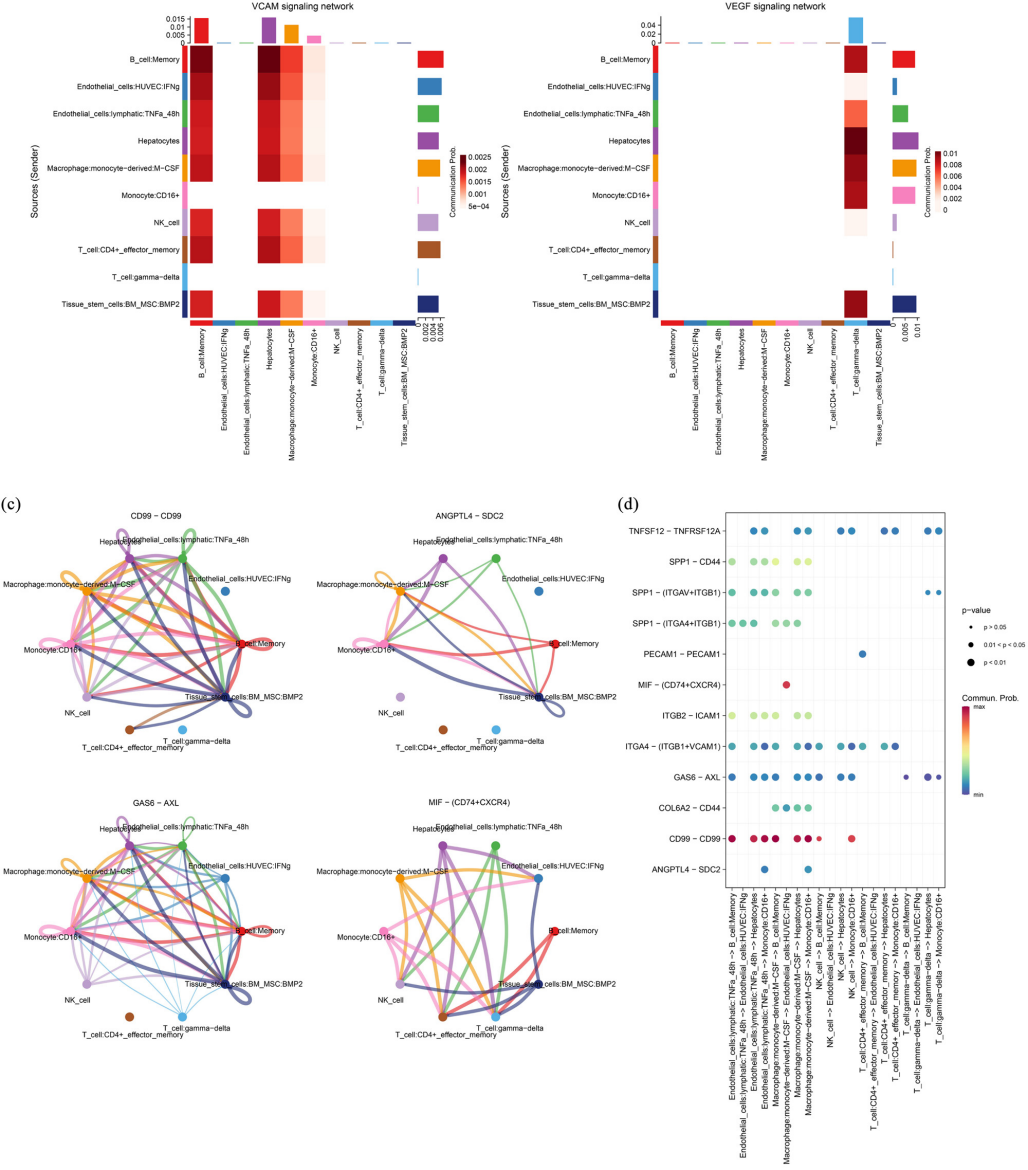
(a) The mutual intensity/probability map of ten kinds of cells. The circles of various colors on the periphery represent the number of cells. The more cells, the larger the circle. The cells that emit arrows express ligands, and the cells that receive arrows express receptors. The more ligand–receptor pairs, the thicker the line. (b) A heat map of cell interactions mediated by a single signaling pathway or ligand–receptor. The vertical axis is the set of cells that send the signal, and the horizontal axis is the set of cells that receive the signal. The color of the heat map represents the signal intensity, and the upper and right bars represent the cumulative value of the intensity. (c) The contribution of each ligand–receptor pair to the signal pathway and the results are represented by a chord diagram. The greater the contribution, the thicker the line. (d) Contribution of all ligand–receptor pairs to all signaling pathways. The results are displayed in a bubble chart. The closer the bubble color is to red, the greater the contribution value. The diameter of the bubble represents the probability, and *P* < 0.05 is statistically significant.

To analyze the ligand–receptor interactions between various cell types in the cancer microenvironment, we used the ligand–receptor database CellChatDB to estimate cell-to-cell interaction scores. First, we analyzed the number of cell interactions for the 12 cell types previously identified by BRCC. We found that a total of ten cell types had ligand–receptor relationships, and all ten cell types had some degree of interaction with each other. T_cell:CD4+_effector_memory was ligand–receptor interactions in the cell with the highest number of cells, followed by Endothelial_cells:lymphatic:TNFa_48h, and T_cell:gamma-delta was the cell with the lowest number of cells in the ligand–receptor interaction (Figure S3). In analyzing the strength of cellular interactions, we found that different cell types interacted with each other to some extent, with immune cells interacting more strongly with other cell types, such as T_cell:CD4+_effector_memory, NK_cell, and T_cell:gamma-delta, with NK_cell having a lower number of cells in BRCC but interacted with other cell types with very high intensity, and Endothelial_cells:HUVEC:IFNg was lower in both the number of cell types interacted with and the intensity of the interaction (Figure 4a). To explore the signaling pathways acting in cellular interactions for the above ten cell types, we used the computeCommunProbPathway function of the R package CellChat to predict the signaling pathways acting in BRCC cellular communication, and the results are presented in a heat map, where we found that the CD99 signaling network, vascular endothelial growth factor (VEGF) signaling network, and GAS signaling network were activated, among which CD99 signaling network and GAS signaling network were critical signaling pathways in BRCC cell interactions, and the high expression of B_cell:Memory was associated with the activation of several signaling pathways, such as CD99 signaling network, growth arrest-specific protein (GAS) signaling network, and vascular cell adhesion molecule (VCAM) signaling network (Figure 4b). Subsequently, to further explore the contribution of each ligand–receptor to the overall signaling pathway, we calculated the contribution of each ligand–receptor to the overall signaling pathway using the extractEnrichedLR function of the R package CellChat and selected the ligand–receptor with the largest contribution to the signaling pathway for presentation. We found that the Tissue_stem_cells:BM_MSC:BMP2 ligand makes a significant contribution to the activation of multiple signaling pathways, including ligand–receptor pairs CD99–CD99, GAS6–AXL, and MIF–(CD74-CXCR4), with the highest contribution in ITGA4–(ITGB1 + VCAM1). The ligand with the highest contribution is T_ cell:CD4+_effector_memory, and the highest contributing ligand in the VEGF-A–VEGF-R signaling pathway was T_cell:gamma-delta (Figure 4c). Finally, we used the netVisual_bubble function of the R package CellChat to infer all interactions between cell groups, the results of which are shown in bubble plots. We found that the ligand–receptor CD99–CD99 is closely associated with intercellular interactions in several cell types of BRCC, and that the TNFSF12–TNFRSF12A signaling pathway, the GAS6–AXL signaling pathway, and the ITGA4–(ITGB1 + VCAM1) signaling pathway also have important roles in intercellular interactions in BRCC (Figure 4d).

## 4 Discussion

BRCC is a rare type of kidney cancer with a severe disease and poor prognosis [22]. The main treatment for BRCC is still a complete surgical resection of the tumor [23]. This includes nephron-sparing surgery (NSS), radical nephrectomy, and physical ablation [24]. Early and appropriate surgical intervention is important for the prognosis of BRCC patients, but there is no consensus on whether to perform a first- or second-stage surgical resection. In an analysis of 71 patients with concurrent bilateral renal cancer, Blute et al. [25] found no statistically significant differences in local recurrence rates, metastasis rates, and tumor-free survival in this group of patients with concurrent bilateral renal cancer compared to unilateral sporadic renal cancer. Becker et al. [26] concluded that the staged surgical option is less of a blow to renal function and, therefore, recommended staged surgery for bilateral renal cancer. There is no consensus on whether the tumor origin of BRCC is the same, and determining whether left- and right-sided ccRCC are of the same origin is an important guide to their clinical management. Based on our analysis of BRCC scRNA-Seq data, based on t-SNE and UMAP clustering and cell annotation results, and comparing the percentage of cells of each cell type in left- and right-sided ccRCC, we concluded that the left- and right-sided ccRCC were of the same tumor origin (Figure 1a–e).

Nonsurgical treatment as an adjunct to BRCC treatment may improve the prognosis of patients with advanced BRCC to some extent and prolong their long-term life expectancy [27]. Ansari et al. [28] found that preoperative administration of sunitinib resulted in the downgrading of limited high-grade primary renal tumors, creating the conditions for NSS in BRCC. The above treatments have to some extent increased the chance of surgery for BRCC patients and prolonged the long-term survival of patients with bilateral renal cancer, but they are all inevitably resistant to late treatment, and RCC is a solid tumor with poor sensitivity to chemotherapy. The above problems are related to the intra- or intertumoral heterogeneity of RCC. It is now thought that different single-cell subpopulations with genetic heterogeneity may evolve during cancer development, that there are dynamic interactions between cancer cells, and that this potential intratumoral heterogeneity is the main reason why some cells in the tumor are not sensitive to targeted therapy [29]. In this regard, to investigate the inter- and intratumoral heterogeneity of BRCC, we investigated the tumor microenvironment of left- and right-sided ccRCC and BRCC integrated by the R package harmony. T cells accounted for the highest number of cells in the left-sided ccRCC, while epithelial cells accounted for the highest number of cells in the right-sided ccRCC. In the analysis of the BRCC tumor microenvironment, we found that the largest proportion of cells was T cells, which may be related to the fact that the number of cells in the left-sided ccRCC was higher than the number of cells in the right-sided ccRCC. When further exploring the malignancy score and stemness score of left- and right-sided ccRCC, there were also significant differences between the two, with left-sided ccRCC being more malignant and having a better immune response than right-sided ccRCC (Figure 2a–f). The above analysis reveals to a certain extent the heterogeneity of BRCC and provides ideas for its targeted therapy and immunotherapy.

Studies have concluded that the vast majority of BRCC is genetically related and that genetically related kidney cancers are significantly more likely to present as bilateral multicentric tumors than non-genetically related kidney cancers, such as classical genetically related renal cancers like von Hippel–Lindau syndrome and hereditary papillary renal cancer [30]. The kidney cancer sample in this study was sporadic bilateral kidney cancer with clear cell carcinoma pathology on both sides. Current studies have shown that there are significant differences in the genetic characteristics, pathogenesis, biological behavior, and treatment between sporadic bilateral renal cancer and hereditary bilateral renal cancer [31]. Dal Cin et al. [32] showed that genetic alterations in bilateral renal carcinomas correlated with pathological histological types, such as chromosomal triploid associated with pRCC, deletion of the short arm of chromosome 3 associated with non-pRCC, and combined Y chromosome and deletion of chromosome 1 associated with eosinophilic tumorigenesis. In addition, there was variability in karyotype between different tumors within the same kidney and between tumors in bilateral kidney. In conclusion, the mechanism of development of bilateral sporadic renal carcinoma is unclear. Here, we have explored the characteristics of BRCC cell developmental trajectories and the role of intercellular communication by analyzing BRCC scRNA-Seq and have partially elucidated the relevant molecular mechanisms in BRCC development and progression. We found that BRCC developmental progression is mainly associated with two cell types, Endothelial_cells:lymphatic-derived and Macrophage:monocyte-derived, which are active in the tumor progression of BRCC (Figure 3c–e). The Endothelial_cells:lymphatic is the main structure that forms the wall of the lymphatic vessels [33] and are involved in physiological processes, such as maintaining fluid balance and regulating lymphocyte recirculation and the immune response [34]. The lymphatic vasculature is the main structure of the lymphatic vessel wall. It has been found that the process of lymphangiogenesis is mainly the migration of lymphatic endothelial cells from existing lymphatic vessels and the formation of new lymphatic vessels, which is closely related to tumor metastasis [35,36,37,38]. Macrophages are composed of monocytes, macrophages, and their precursor cells and are a highly heterogeneous and plastic class of immune cells with M1 and M2 phenotypes [39]. Under the mediation of the microenvironment, tumor-infiltrating macrophages undergo M2 polarization and participate in tumor progression by blunting antitumor immunity, stimulating vascular lymphangiogenesis, and promoting cell invasion and metastasis [40,41,42]. The effect of mesenchymal stem cells is to regulate the self-renewal, quiescence, and mobilization of hematopoietic stem cells and promote neovascularization in tumor tissue. RCC is the most active solid tumor in terms of angiogenesis, and we hypothesize that this may be related to the high expression of Macrophage:monocyte-derived and the occurrence of M2-type polarization. In the process of exploring intercellular interactions in BRCC, we found that immune cells were active in intercellular interactions, both in terms of the number and intensity of interactions with other cell types, such as T_cell:CD4+_effector_memory, NK_cell and T_cell:gamma-delta immune cells. This suggests that the tumor immune microenvironment has a strong influence on the development of BRCC and may be more responsive to immunotherapy (Figure 4a and b). In exploring the mode of action of γδ T cells in BRCC cell interactions, we found that they may act through the VEGF and macrophage wandering inhibitory factor signaling network and analyzed that the ligand–receptor pairs they act on are vascular endothelial growth factor–vascular endothelial growth factor receptor1 (VEGF–VEGFR1), MIF–(CD74–CXCR4), and GAS6–AXL (Figure 4c and d), suggesting a pro-carcinogenic role for γδT cells in the development of BRCC. VEGF–VEGFR1 is an extremely important signaling pathway in tumor neovascularization, and VEGF binding to VEGFR1 promotes vascular endothelial cell mitosis by activating phospholipase C and stimulating second messenger formation, inducing fibrinolytic zymogen degradation, participating in basement membrane degradation and extracellular protein hydrolysis, and increasing vascular permeability. VEGFR1 activation can regulate the interaction between endothelial cells and endothelial cells and endothelial cells and basement membrane, and it can also promote microvascular lumen formation [43,44]. GAS6–AXL is also an important signaling axis in tumor neovascularization. Growth arrest-specific protein 6 (GAS6) is a secreted vitamin K-dependent protein that binds to the receptor AXL and promotes tumor neovascularization by regulating endothelial cell function, the activation status of integral proteins, and the expression of pro-angiogenic factors [45,46,47,48]. In conclusion, BRCC promotes its tumor progression through complex intercellular interactions by activating cancer-related signaling pathways, such as cell proliferation, cell differentiation, angiogenesis, immunity, and tumor metastasis.

This study also has some flaws, as we annotated BRCC cells with reference to HPCA, and we found a small number of hepatocytes in this sample, but this did not occur when they were annotated with a custom cell type marker (Table 1) (Figure 1f and g). We speculate that this may be related to an error in the cell type marker built into the HPCA. As HPCA is the definitive cell type marker database, our custom cell type markers were not able to annotate cell subtypes, whereas HPCA was able to annotate cell subtypes better and its annotated cell types were generally more reliable and comprehensive. Therefore, we used this annotation data in a series of subsequent analyses. Second, the present sample is a small sample of single-cell sequencing data and the conclusions drawn are not very reliable. More single-cell sequencing data are needed to further confirm these conclusions, but the incidence of sporadic bilateral kidney cancer is very low in clinical practice and bilateral kidney cancer is mostly a genetically related disease. Sporadic BRCC samples are difficult to obtain, fewer samples are available for single-cell sequencing, and large sample studies require mutual assistance between medical institutions to achieve.

Through the exploration of BRCC scRNA-Seq data, we have preliminarily elucidated the tumor origin, complex intratumoral environment, cell developmental trajectory features, and cell-to-cell interaction patterns of BRCC, providing ideas for further study of the molecular mechanisms of its pathogenesis and immunotherapy.

## Supplementary Material

Supplementary Figure
