# Supplementary material for: Single-cell transcription analysis reveals the tumor origin and heterogeneity of human bilateral renal clear cell carcinoma
Source: Open Life Sci. 2023 Feb 9;18(1):20220569. doi: 10.1515/biol-2022-0569 (PMC9922059; doi:10.1515/biol-2022-0569)
Supplement: Supplementary Figure [file biol-2022-0569-sm.pdf]

Supplementary material

1. Cell quality control and data integration (Figures S1, S2, and S3)

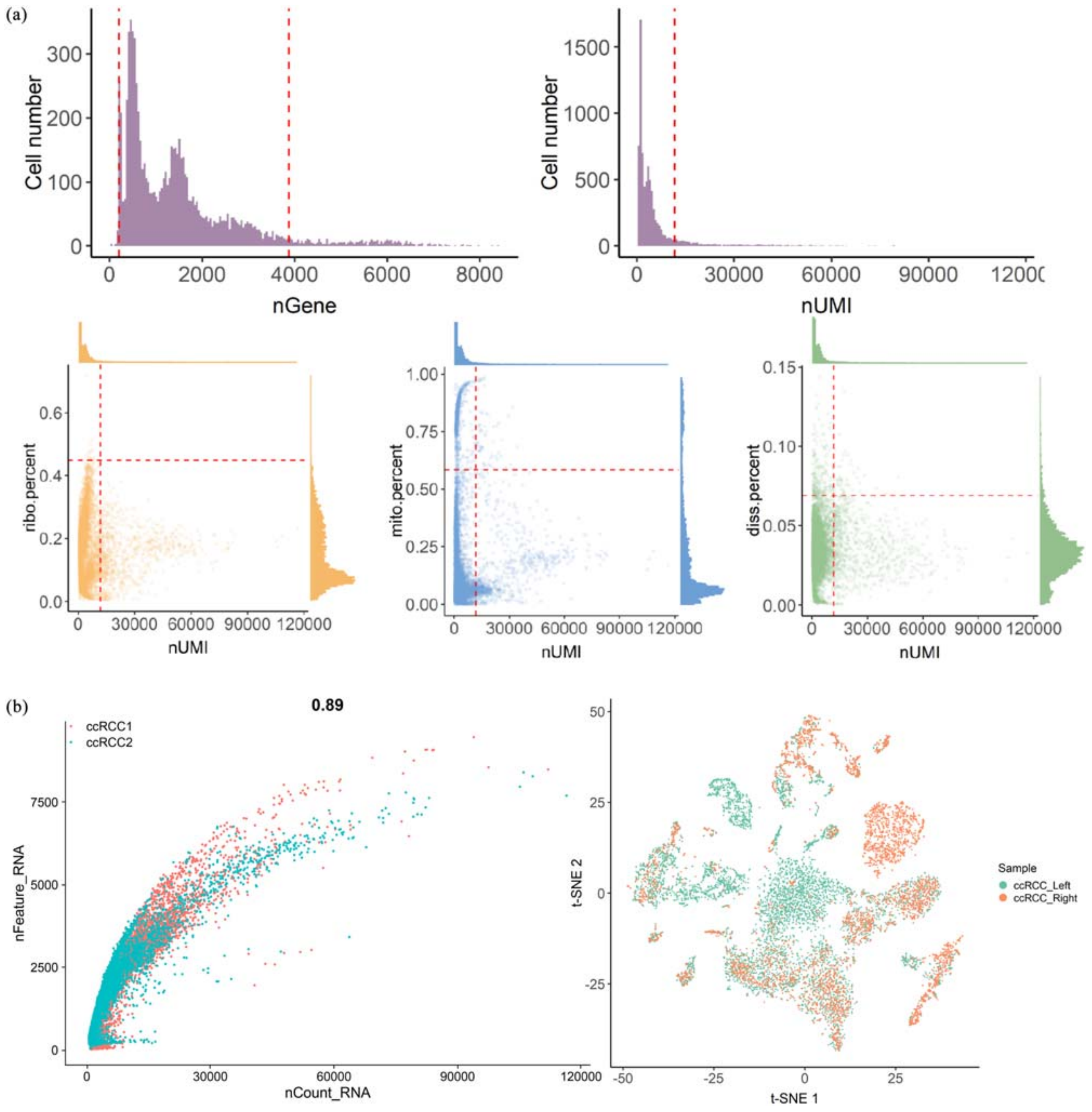

Figure S1: Quality control (QC) of the scRNA-seq data. (A)QC of BRCC scRNA-seq data. nFeature, number of genes; nCount, unique molecular identifiers (UMIs). (B)Scatter diagram of sample distribution of Left and Right ccRCC. (C)The scRNA-Seq data of left and right ccRCC were integrated by PCA、tSNE and UMAP respectively. (D,E)the number of UMIs and Doublet score estimation of Right、Left ccRCC scRNA-seq data. Notes：ccRCC1：Right ccRCC, ccRCC2：Left ccRCC.

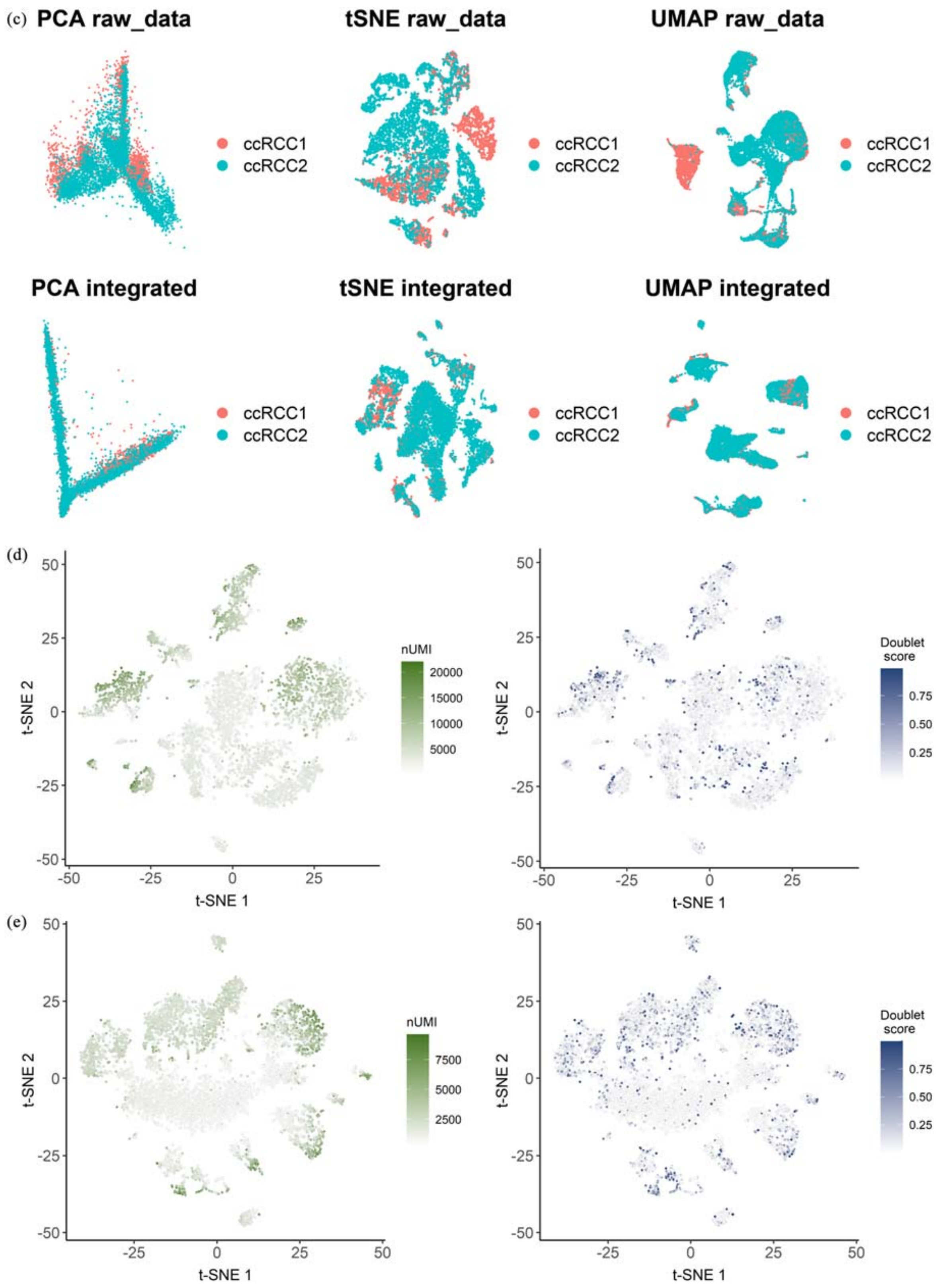
(c)
PCA raw_data
tSNE raw_data
UMAP raw_data
ccRCC1
ccRCC2
PCA integrated
tSNE integrated
UMAP integrated
(d)
nUMI
20000
15000
10000
5000
Doublet score
0.75
0.50
0.25
t-SNE 1
t-SNE 2
(e)
nUMI
7500
5000
2500
Doublet score
0.75
0.50
0.25
t-SNE 1
t-SNE 2

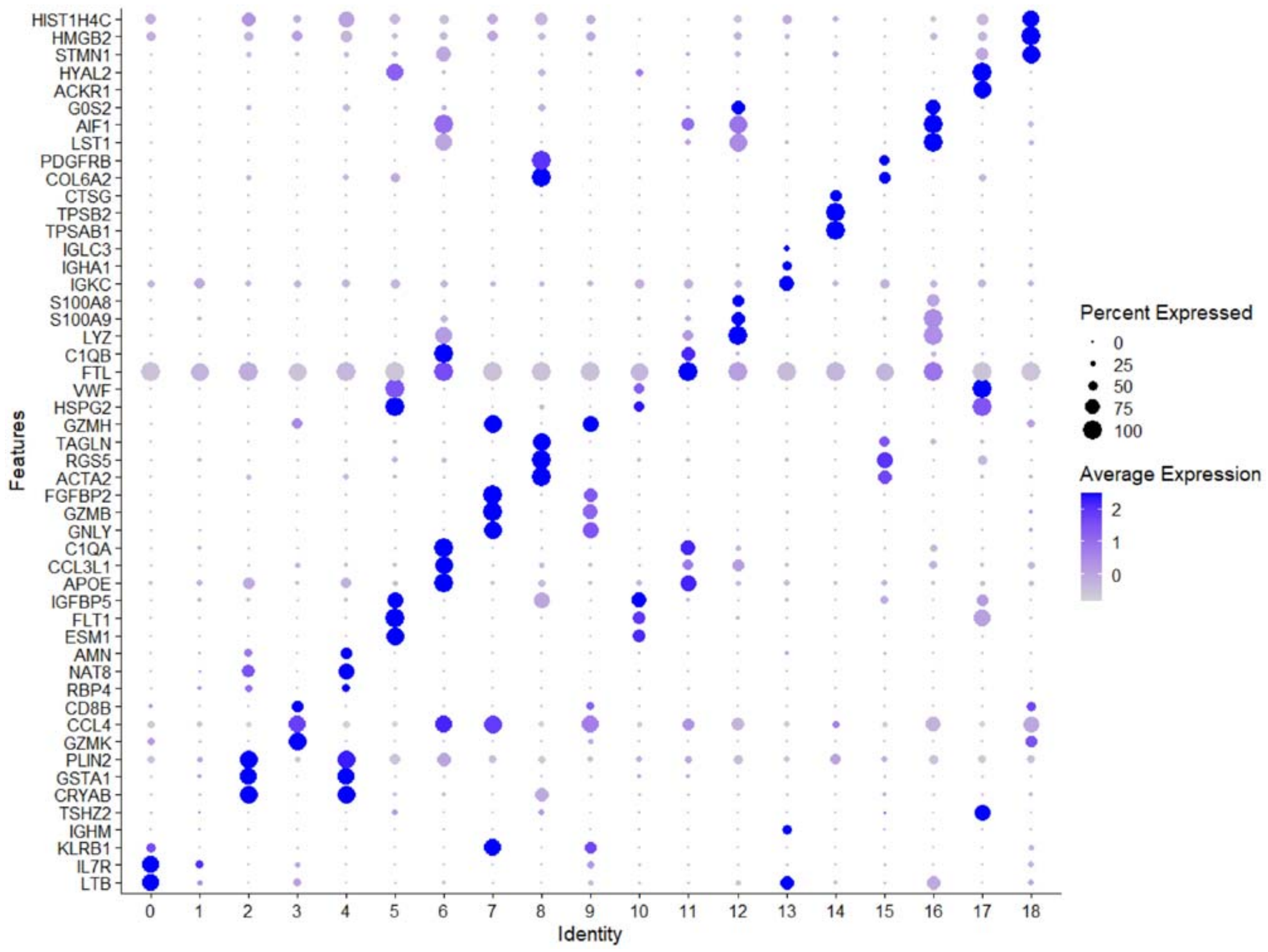

Figure S2: Bubble plots of T03 gene expression in each cell cluster.

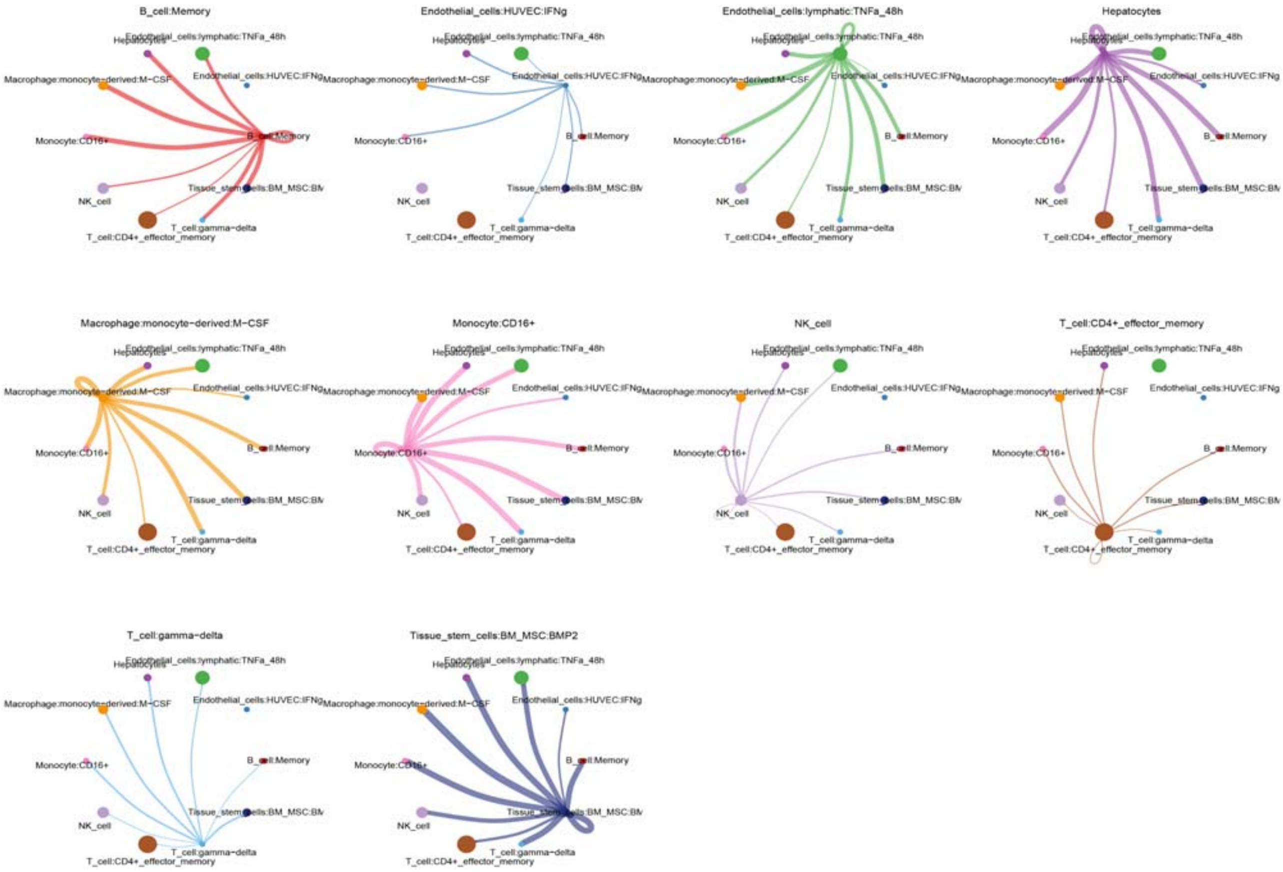

Figure S3: The interaction intensity/probability diagram between a single cell and other cells, the circles of various colors represent the number of cells, the more cells, the larger the circle, the cells that emit arrows express ligands, and the cells that receive arrows to express receptors, the more ligand-receptor pairs, the thicker the line.
